# Farmers′ use of climate change adaptation strategies and their impacts on food security in Kenya

**DOI:** 10.1016/j.crm.2023.100495

**Published:** 2023

**Authors:** Girma Gezimu Gebre, Yuichiro Amekawa, Asmiro Abeje Fikadu, Dil Bahadur Rahut

**Affiliations:** a Faculty of Environment, Gender and Development Studies, Hawassa University, Hawassa, Ethiopia; b The Japan Society for the Promotion of Science (JSPS) Postdoctoral Research Fellowship Program, Ritsumeikan University, Kyoto 603-8577, Japan; c College of International Relations, Ritsumeikan University, Kyoto, Japan; d Department of Agricultural Economics, Debre Tabor University, Debre Tabor, Ethiopia; e Department Agricultural and Resource Economics, Kyushu University, Japan; f Asian Development Bank Institute, Tokyo, Japan; g International Maize and Wheat Improvement Center, Mexico

**Keywords:** Adaptation strategies, Climate change, Food security, Smallholder farmers, Kenya

## Abstract

Climate change threatens the sustainability of food production among farmers in Kenya who depend on rain‐fed agriculture. To minimize the negative impacts of climate change, farmers have sought to adopt different adaptation strategies. This study investigates factors influencing farmers’ choice of climate change adaptation strategies and associated effects on their food security in Kenya using data collected from 540 farmers from six counties. A multivariate probit, censored least absolute deviation (CLAD), and propensity score matching (PSM) models were employed to identify the determinants in the farmers’ choice of climate change adaptation strategies, the number of adaptation strategies adopted, and the effect of climate change adaptation strategies on their food security, respectively. Results show that planting drought-tolerant crop varieties (55%), growing diversified crops (34%), growing early maturing crops (22%), and diversifying the sources of household income (18%) were the four major adaptation strategies used by the farmers in the study area. Younger farmers and those with higher education levels are more likely to use these climate change adaptation practices. The number of adaptation strategies used was positively associated with male farmers, education level, family size, land size, farm income, extension contact, training, and information access. The farmers who adopt one adaptation strategy have higher food security status (approximately 7–11%) than those who do not. If they adopt two adaptation strategies, their food security status increases by approximately 11–14%; if they adopt three adaptation strategies, their food security status increases by nearly 12–15%; and if they adopt four adaptation practices, their food security status increases by about 14–18%, compared to those who do not adopt any strategy. Thus, the farmers’ climate change adaptation practices have positive food security effects in Kenya according to the number of adaptation strategies adopted.

## 1 Introduction

Climate change is a significant and growing threat to the global agricultural system (Musafiri et al., 2021), food security (De Pinto et al., 2019, Muchuru and Nhamo, 2019), and nutritional outcomes (De Pinto et al., 2019). It challenges the 2030 agenda of Sustainable Development (Inter-governmental Panel on Climate Change [IPCC], 2022), which was adopted by the United Nations General Assembly on September 25, 2015, with an objective to “end hunger and ensure access by all people, in particular, the poor and people in vulnerable situations, including infants, to safe, nutritious and sufficient food all year round” (FAO, 2017). Climate change will not only threaten the productivity of the world‘s agricultural systems and associated food security but also negatively affect other ecosystems and their services to humankind (IPCC, 2022; De Pinto et al., 2019). According to IPCC, 2014, the average global temperature has increased over the 20th century by approximately 0.6 °C. A report from the IPCC (2018) shows that half a degree of warming matters a lot. As part of the historic Paris Agreement on climate change, the countries are committed to keep global warming well below 2˚C (3.6˚F) above pre-industrial levels while trying to limit a temperature rise to 1.5˚C (2.7˚F) (Levin, 2018). Based on a request by governments, scientists from the IPCC estimated how the impacts of a 1.5˚C temperature rise differ from those of 2˚C. Their finding shows that the world will face severe climate impacts even with 1.5 degrees of warming, and the consequences of a 2 °C warmer world will be far greater than that of a 1.5 °C warmer world (e.g., the average global crop yield loss by 3% will increase to 7%; the decline in global GDP by 3% will increase to 5%) (IPCC, 2018). However, the world is not on track to meet either target (World Bank, 2019a). *Without substantial measures that address the challenges caused by increasing temperatures and the increased fre*quency and intensity of extreme weather events, agricultural productivity losses are expected to reduce past gains from technological and management improvements (De Pinto et al., 2019).

Disasters triggered by weather- and climate-related hazards cost the global economy US$320 billion in losses in 2017 alone (World Bank, 2019a). Repeated disasters slow the development of infrastructure systems and reduce the productivity of local economies (World Bank, 2019b). Analysis by the United Nations Development Program (UNDP, 2008) shows that the impacts of climate change will add 600 million people worldwide to the number already facing malnutrition and will increase the number facing water scarcity by 1.8 billion by 2080. Climate change disproportionately affects vulnerable populations living in agricultural communities in developing countries (Maskrey et al., 2007, Rahut and Ali, 2017, Muchuru and Nhamo, 2019, Aryal et al., 2021) and is expected to affect many more people in more areas in the future (De Pinto et al., 2019). The worst-hit areas will be underdeveloped economic regions (De Pinto et al., 2019), such as sub‐Saharan Africa (Hadebe et al., 2016, Muchuru and Nhamo, 2019), where food security is already a major problem, and populations are highly vulnerable to climatic and other shocks (Drammeh et al., 2019, Kabubo-Mariara and Mulwa, 2019, Gebre and Rahut, 2021).

In recent decades, climate change has particularly intensified in sub‐Saharan Africa (IPCC, 2007; Franklin et al., 2021) and poses a threat to the sustainability of the food production system among small‐scale rural communities that are dependent on rain‐fed agriculture (Nelson et al., 2009, Fadina and Dominique, 2018, Atube et al., 2021). IPCC predicts that, by 2050, crop productivity in sub‐Saharan Africa will have declined by 5% for maize, 14% for rice, and 22% for wheat, pushing a large number of already vulnerable people, who depend on agriculture for their livelihoods, deeper into poverty and food insecurity (IPCC, 2018). IPCC also predicts decreased food availability by 500 cal less (a 21% decline) per person in 2050 and a further increase in the number of malnourished children by over 10 million, to a total of 52 million in 2050 in sub‐Saharan Africa alone (IPCC, 2018). The World Bank reported similar concerns that, without concrete climate and development action, up to 86 million people could become climate migrants within their own counties in the sub-Saharan African region by 2050 (World Bank, 2021). Consequently, individuals, families, and even whole communities will be forced to seek more viable and less vulnerable places to live (Rigaud et al., 2018). Of the projected 86 million people, a large proportion (38.5 million) will be from the Lake Victoria basin countries, such as Kenya, Tanzania, and Uganda (World Bank, 2021).

The adverse effects of climate change in East African countries are more severe mainly due to the interaction of multiple factors, including high population growth, extreme poverty, poor infrastructure, overdependence on rain-fed agriculture, poor availability and quality of meteorological data, and knowledge gaps (Agidew and Singh, 2018, IPCC, 2007, IPCC, 2014; Kabubo- Mariara & Mulwa, 2019; Drammeh et al., 2019, Aryal et al., 2021, Gebre and Rahut, 2021). The impacts of climate change make it imperative to explore available adaptation strategies and their constraints for adoption to offset its current and future adverse effects on the region. Climate adaptation-based development approaches can enable these countries to diversify and become less reliant on sectors that are more vulnerable to climate change effects, while increasing the capacity for people to withstand shocks. This approach also makes more resources available to countries, communities, and people to minimize the impact of risks (World Bank, 2010). Early adaptation actions could promote development by reducing risks and costs associated with asset losses from climate-related disasters, reducing infrastructure repair costs, and creating new opportunities (World Bank, 2019a).

Agricultural adaptation strategies to climate change take a wide range of forms that include: planting drought-tolerant crops, early planting, crop diversification, rainwater harvesting, market responses, such as income diversification and credit schemes, developing meteorological forecasting capability, and improving agricultural markets and information provision (Kabubo-Mariara & Karanja, 2007; Hassan and Nhemachena, 2008, Deressa et al., 2009; Lybbert & Sumner, 2012; Mburu et al., 2015; Mulwa et al., 2017; Shisanya and Mafongoya, 2016, Rahut and Ali, 2017, Ali and Erenstein, 2017, Amare and Simane, 2017, Fadina and Dominique, 2018, De Pinto et al., 2019, Kabubo-Mariara and Mulwa, 2019, Aryal et al., 2020, Bairagi et al., 2020, Kogo et al., 2020, Aryal et al., 2021, Atube et al., 2021; Franklin et al., 2021; Gebre & Rahut, 2021).

In Kenya, climate change affects weather patterns and causes shifts in seasons with serious repercussions, such as declining food production and productivity for communities and farming households (Mburu et al., 2015, Kabubo-Mariara and Mulwa, 2019, Aryal et al., 2021; Musafiri et al., 2021). This worsens the food security situation of smallholders and subsistence farmers in the country, making it difficult for their household members to obtain enough food in their daily life (Mburu et al., 2015, Kabubo-Mariara and Mulwa, 2019, Kogo et al., 2020, Gebre and Rahut, 2021, Ndiritu and Muricho, 2021). The agricultural sector plays a significant role in Kenya’s economy, contributing to about 30% of GDP and 56% of employment (World Bank, 2019b). To reduce vulnerability to climate change, farmers in Kenya use different adaptation strategies, such as planting drought-tolerant crop varieties, altering sowing time, shifting to new crops (e.g., early maturing crops), use of water harvesting technologies and irrigation, crop rotation, crop diversification, and income diversification (Mburu et al., 2015, De Pinto et al., 2019, Kabubo-Mariara and Mulwa, 2019, Kogo et al., 2020, Aryal et al., 2021; Franklin et al., 2021; Musafiri et al., 2021). These adaptation practices typically reduce the risks of exposure to natural hazards associated with climate change and the severity of damages caused. Thus, farmers using climate change adaptation strategies are more likely to be food secure in comparison to those not adopting any strategy.

Kenya has experienced extreme weather events, such as increased erratic rainfall, intra- seasonal dry spells, incidences of flooding, high temperatures, and a higher frequency of droughts, causing enormous damage to the crops and livelihoods of smallholder farmers (Aryal et al., 2021; Musafiri et al., 2021). It is anticipated that these adversities will increase due to climate change. The significance of climate change adaptation strategies is crucial given the importance of agriculture for food security and the economy and rural livelihoods. Nonetheless, not all farmers adopt such practices. Moreover, some farmers may use one adaptation practice alone, while others may use two or more adaptation practices in combination. Thus, even among the adopters, there may exist differences in the benefits derived from the use of adaptation practices (Ali & Erenstein, 2017).

Numerous studies have been conducted on the determinants of climate change adaptation in Kenya (e.g., Bryan et al., 2013, Jairo & Korir, 2019; Mutunga et al., 2020, Aryal et al., 2021; Musafiri et al., 2021). However, studies regarding the impact of adopting climate risk adaptation strategies on the food security of farmers in Kenya are scarce. Few studies measured the effects of climate change adaptation on food security by dividing farmers (farm households) into adapters and non-adapters, yet without considering the number of adaptation practices adopted by farmers (e.g., Kabubo-Mariara and Mulwa, 2019, Ndiritu and Muricho, 2021). Additionally, no studies have tried to identify determinants of the number of adaptation strategies farmers adopt in Kenya. Identifying determinants of the number of adaptation strategies would provide clues as to what factors work as stronger leverage over others and vice versa, regarding how many climate change adaptation strategies farmers adopt. Furthermore, the present study argues that farmers who adopted one adaptation strategy could have higher food security status than non-adopter farmers; likewise, those who adopted more adaptation strategies could have higher food security status than those who adopted less. Hence, this study considers the differential impact of climate change on food security according to the number of adaptation strategies adopted.

The contribution of this paper is threefold: first, it uses a multivariate probit to identify the determinants in farmers’ choice of adaptation strategies simultaneously (specifically, planting of drought-tolerant crop varieties, crop diversification, use of early maturing crop varieties, and income diversification); second, it identifies the determinants of the number of strategies used by farmers; and third, it assesses the effect of these adaptation strategies on farmers’ food security by taking into account the number of adaptation practices adopted.

The rest of the paper has been structured in the following manner: Section 2 discusses the conceptual and empirical frameworks; Section 3 describes the data and variables used in the models; Section 4 presents the empirical results and discussion; finally, Section 5 presents the conclusion and policy implications.

## 2 Conceptual and empirical frameworks

### 2.1 Conceptual framework

**Fig. 1:**
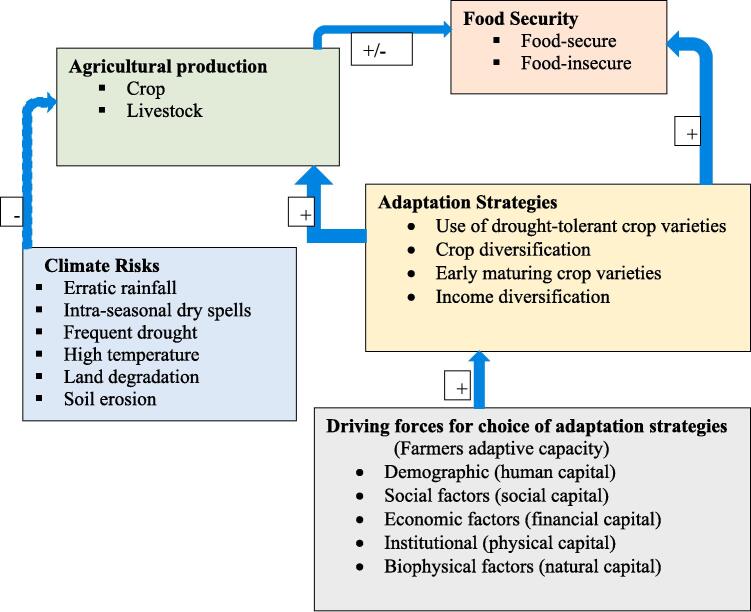
Conceptual framework of the study. *Note that the arrows and the*±*signs indicate the expected direction of effects on the corresponding variables or indicators; the dashed line indicates a potential impact.*

This study conceptualizes the link between climate change adaptation and food security based on a series of causal relationships (Fig. 1). Climate risks, including extreme weather events, involve the possibility of the occurrence of various natural hazards, such as erratic rainfall, intra-seasonal dry spells, frequent drought, high temperature, land degradation, and soil erosion. Such climate adversities can negatively affect agricultural production (crop and livestock), and hence, the food security of farm households (Eitzinger et al., 2018, Gebre and Rahut, 2021, IPCC, 2014, Kabubo-Mariara and Mulwa, 2019, Ndiritu and Muricho, 2021). To reduce the adverse impacts of climate risks on their food security, the farmers will adopt several adaptation strategies (Ali and Erenstein, 2017, Amare and Simane, 2017, Aryal et al., 2020).

Farmers’ adoption (choice) of adaptation strategies against climate risks depends on multiple factors, including farmers’ demographic (human capital), socioeconomic (social and financial capital) characteristics as well as other institutional and biophysical factors (physical and natural capital) (Aryal et al., 2020; 2021; Atube et al. 2021; Franklin et al. 2021; Gebre & Rahut, 2021). When the adopted adaptation strategies are adequate and effective, it will increase the agricultural production of farm households and better ensure their food security, and vice versa. Income diversification adaptation strategy can also positively influence their food security status by improving their financial capital base to purchase foods (Zakari et al., 2022).

### 2.2 Empirical framework

In Kenya, farmers adopt different climate change adaptation strategies to ensure food security. Therefore, we first employed a multivariate probit model to identify the choice determinants of multiple climate adaptation strategies, including the use of drought-tolerant crop varieties, crop diversification, early maturing crop varieties, and income diversification. Employing other discrete choice models, such as univariate logit and probit, are not appropriate in this case as they may generate biased estimates. Greene (2003) notes that these models are based on the assumption of the independence of error terms pertaining to the different adaptation practices adopted by farmers. Univariate models could also exclude critical information about interdependence and simultaneous adoption decisions (Greene, 2019). Possible complementarities (positive correlation) and substitutabilities (negative correlation) could also occur between various adaptation strategies used by farmers (Greene, 2019). In the study sites, farmers are more likely to adopt multiple adaptation strategies simultaneously in order to reduce climate change risks related to their food security. Therefore, it is highly likely that the decision to adopt one strategy can influence the adoption of multiple other strategies. Applying a multivariate probit model in this condition yields unbiased and efficient estimates (Wooldridge, 2012, Greene, 2019). This also reduces the possibility of observing the limited adoption of one or a few adaptation strategies due to the non-adoption of other complementary strategies (Aryal et al., 2020).

Checking potential endogeneity and multicollinearity is critically important for applying a multivariate probit. An endogeneity possibly occurs through (i) simultaneity, (ii) omitted variables, or (iii) measurement errors, leading to inconsistent estimates (Wooldridge, 2010). However, the distinctions among these (three) possibilities are not easily observed (Deaton, 1995). In our study, an explanatory variable could be jointly determined with the decision to adopt an adaptation strategy in the model (i.e., the response variable) (Abdulai & Huffman, 2014). To check for potential endogeneity, we used the approach suggested by Rivers & Vuong (1988). We specified the potential endogenous response variable as a function of all other explanatory variables in the model, including a set of instrumental variables correlated with the endogenous variable but excluding error terms. After regressing the endogenous variable against all explanatory variables (including instrumental variables), we calculated a residual term from the regression results of the endogenous variable. Then, we included the residual term as an additional explanatory variable in the model. The estimates obtained from this model are consistent (Wooldridge, 2010). However, in the empirical estimation, it is not possible to obtain a good instrumental variable (Aryal et al., 2021). Considering the limitation, as Gujarati & Dawn (2009) suggested, the present study tested for endogeneity using the residual term as the sole additional explanatory variable.

In our study, the distance from a farm to the agricultural extension service center can be taken as an instrumental variable, assuming that it affects farmers’ access to information on their possible adaptation strategies, but it does not influence their selection of individual adaptation strategies (Aryal et al., 2021). Multivariate probit results from testing for endogeneity confirm no endogeneity in the present study. We applied a condition index to test for the potential multicollinearity among explanatory variables in the multivariate probit model. If the value of the condition index is less than 30, it indicates no serious problem of multicollinearity among the explanatory variables used in a multivariate probit analysis (Belsley et al., 2005).

We consider a risk-averse farmer Fi who opts for a number of climate change adaptation strategies (Si). We assume that farmers who have opted for climate change adaptation strategies have higher utility levels than those who have not.

(1) $$U\left[F\left(S_{1}\right)\right]>U\left[F\left(S_{0}\right)\right]\;\;\;\;\;\;\;\;\;\;\;\;\;\;\;\;\;\;\;\;\;\;\;\;\;\;\;$$

$U\left[F\left(S_{1}\right)\right]$ and $U\left[F\left(S_{0}\right)\right]$ represent the utility levels of farmers having adopted only one strategy and those of farmers not having adopted any strategy, respectively. We further assumed that farmers adopting two strategies have higher utility levels than those adopting only one strategy, and so on.

(2) $$U\left[F\left(S_{1},\;S_{2}\right)\right]>U\left[F\left(S_{0},\;S_{1}\right)\right]\;\;\;\;\;\;\;\;$$

Second, a censored least absolute deviation (CLAD) model was employed to estimate the number of adaptation strategies adopted by the farmers. The CLAD estimator is a generalization of the least absolute deviation (LAD) estimator. Unlike the standard estimators of the censored regression model, such as Tobit or other maximum likelihood approaches, the CLAD estimator is robust to heteroscedasticity and is consistent and asymptotically normal for a wide class of error distributions. As the CLAD estimator imposes the weakest stochastic restrictions on the error terms, it results in the most precise estimates of the policy effects (Ali & Erenstein, 2017).

Third, a propensity score matching (PSM) method was employed to estimate the effect of the number of adaptation strategies on farmers’ food security in Kenya. The expected treatment effect for the treated population is of primary significance, 1and it is given as

(3) $$\mathrm{ATT}=E\left(\Delta |D=1\right)=E\left(Y_{1}|x,\;D=1\right)-E\left(Y_{0}|x,\;D=1\right)\;\;\;\;\;\;\;\;\;\;\;\;\;\;\;\;\;$$

where ATT is the average treatment effect for the treated, $Y_{1}$ denotes the value of the outcome for adopters of a climate change adaptation strategy, and $Y_{0}$ is the value of the same variable x for non-adopters of the climate change adaptation strategy. As noted above, the major problem with this procedure is that the counterfactual $E\left(Y_{0}|x,D=1\right)$ is not based on empirical observation. Although the difference $\mathrm{ATT}=\left(Y_{1}|x,D=1\right)-E\left(Y_{0}|x,D=0\right)$ can be estimated, it is potentially a biased estimator. In the absence of experimental data, the PSM can be employed to account for this sample selection bias (Dehejia & Wahba, 2002). The PSM is defined as the conditional probability that a farmer adopts a new adaptation strategy with given pre-adoption characteristics (Rosenbaum & Rubin, 1983). To create the condition of a randomized experiment, the PSM employs the unconfoundedness assumption, also known as the “conditional independence assumption,” which implies that once Z is controlled for, a climate change adaptation strategy is random and uncorrelated with the outcome variables (food security in our case). In short, the outcomes are independent of treatment. The PSM can be expressed as:

(4) $$P\left(Z\right)=Pr\left\{D=1|Z\right\}=E\left\{D|Z\right\}\;\;\;\;\;\;\;\;\;\;\;\;\;\;\;\;\;\;\;\;\;\;\;\;\;\;\;\;\;\;\;$$

where D is the indicator for adoption and Z is the vector of pre-adoption characteristics (Abara & Singh, 1993). The conditional distribution of Z given $P\left(Z\right)$ is similar between the adopter and non-adopter groups. After estimating the propensity scores, the average treatment effect for the treated (ATT) can be estimated as:

(5) $$\mathrm{ATT}=E\left\{Y_{1}-Y_{0}|x,\;D=1\right\}=E\left\{E\left\{Y_{1}-Y_{0}|x,\;D=1,\;p\left(Z\right)\right\}\right\}=E\left\{E\left\{Y_{1}|x,D=1,\;p\left(Z\right)\right\}-E\left\{Y_{0}|x,\;D=0,\;p\left(Z\right)\right\}|x,D=0\right\}\;\;\;\;\;\;\;$$

Several techniques have been developed to match adopters with non-adopters of similar propensity scores. The PSM rests on two strong assumptions, i.e., the conditional independence assumption and the common support condition. The conditional independence assumption implies that selection is solely based on observable characteristics and that the researcher observes all variables that simultaneously influence treatment assignment and potential outcomes. (Caliendo & Kopeinig, 2008). The common support assumption implies that for each possible value of *Xi*, there should be individuals in both treatment and control groups for which we can average the outcomes. Implementing the common support condition ensures that any combination of characteristics observed in the treatment group can also be observed among the control group (Bryson et al., 2002).

The most important variable of interest for the PSM is the average treatment effect for the treated (ATT). In our study’s context, ATT is the difference in the outcome of the farmers having used climate change adaptation strategies and similar farmers not adopting. For the PSM estimation of this study, four different matching algorithms, i.e., nearest neighbor matching (NNM), radius matching (RM), kernel-based matching (KBM), and stratified matching (SM) were employed. The matching algorithm helps us to choose and determine the region of common support in a PSM analysis. However, there is no algorithm that dominates in all data situations. Therefore, we employed four types of PSM algorithms commonly used in the PSM analysis to check the level of diversity in the obtained results. After matching for each algorism, the matching quality has to be accessed, and the standard errors and treatment effects have to be estimated. We employed several balancing tests to assess the matching quality, such as checking a reduction in the median absolute bias before and after matching, the value of $R^{2}$ before and after matching, and the p-value of joint significance of covariates before and after matching (Becker and Ichino, 2002, Caliendo and Kopeinig, 2008, Ali and Erenstein, 2017, Rahut and Ali, 2018).

In this study, we used the PSM to estimate the impact of the number of climate change adaptation strategies on the farmers’ food security status. The food security measure/cut-off point was calculated using the Household Food Insecurity Access Prevalence (HFIAP) indicators (Coates et al., 2007; Heady & Ecker, 2012). For each farmer, the Household Food Insecurity Access (HFIA) category variable was calculated using the assigned codes of the degree of food security states in which it fell. Accordingly, four sequential categories of food security states were created (1 = food-secure, 2 = mildly food-insecure, 3 = moderately food-insecure, and 4 = severely food-insecure) according to their most severe response. Each category was calculated by dividing the number of farmers in one category by the total number of farmers in the four categories. Accordingly, 44% of the sampled farmers were identified in the food-secure state, 15% in the mildly food-insecure state, 35.5% in the moderately food-insecure state, and 5.5% in the severely food-insecure state. Additionally, we merged the three food-insecure states (mildly, moderately, and severely) into the food-insecure farmer category and the rest into the food-secure farmer category. Accordingly, the total sampled farmers were halved into the food-secure (56%) and food-insecure (44%) categories. A dummy variable is assigned ‘one’ if the farmer is in the food-secure category and ‘zero’ otherwise. The effect is estimated according to the number of strategies/practices adopted by the farmers. For instance, if one farmer adopts one strategy, the result shows the level of effect on the farmer’s food security status.

## 3 Data and variables description

### 3.1 Survey design and data

**Fig. 2:**
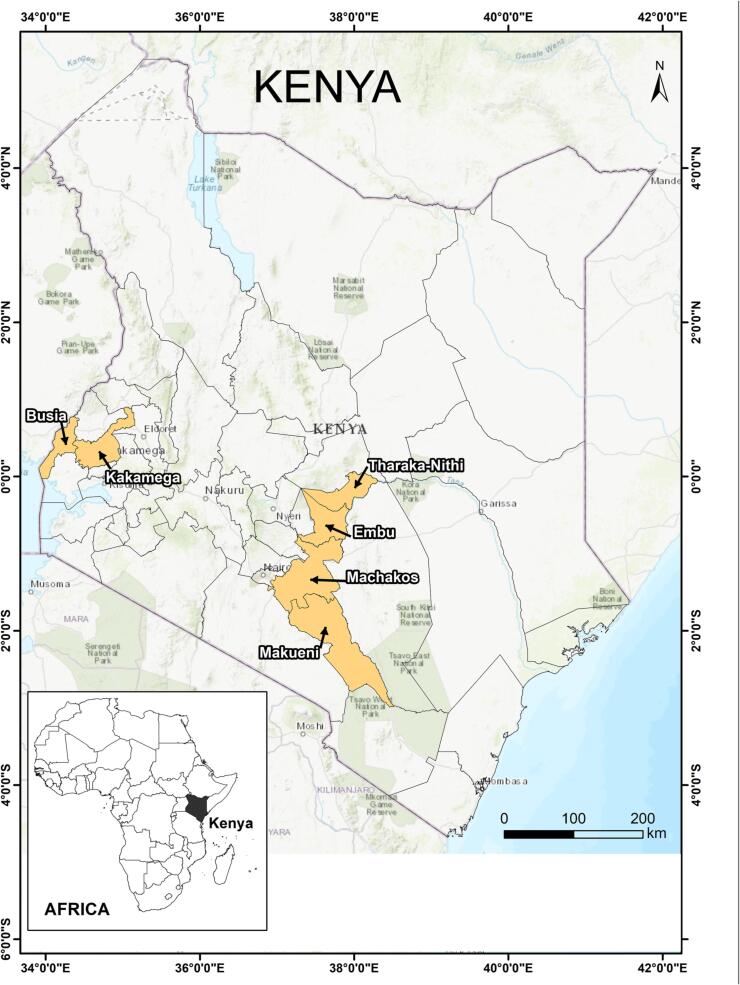
Map the study area. Source: Authors.

The study is based on a set of household survey data collected from November to December 2018. It involved a total sample of 540 farmers from six counties of Kenya, including Makueni, Machakos, Embu, Tharaka Nithi, Kakamega, and Busia (Fig. 2). Those six counties of Kenya adequately represent the climate patterns in Kenya, where climate hazards and shocks are frequently experienced by rural residents in the country’s western, central, and southern regions.

Regarding our sampling method, a multistage, random sampling technique was used in the selection of farmers. The first stage involved the selection of counties under the Feed the Future zones of influence and where major crops, such as maize, is grown, which led to the selection of the six counties. The second stage involved the selection of three major crop farming villages in each country. The criterion for the village selection under the STMA project was that one village holds field demonstrations for drought-tolerant crop varieties, another is its neighboring village, and the third village is distant from the two villages. Finally, at least 30 farmers representing their farming households were randomly selected from each of the three villages, leading to the selection of 90 farmers per county and a total sample of 540 farmers. A semi-structured questionnaire was designed and tested to collect a range of information related to the farmers’ demography, their socioeconomic and agronomic features, and their food security conditions, with consideration for their perceptions of food security status. The questionnaire also captured social networks, institutional arrangements, climate and weather information, and adaptation practices.

### 3.2 Description of the variables

Table 1 presents the summary of descriptive statistics for the variables used in the analysis. The surveyed farmers adopted at least four different strategies to minimize the impact of climate change on their food security: planting drought-tolerant crop varieties (55%), growing diversified crops (34%), growing early maturing crops (22%), and diversifying the sources of their income (18%).

**Table 1:** Description of the variables.

**Variables**	**Description and measurement**	**Mean**
**Dependent Variables**		
Drought-tolerant crop	Dummy, 1 if the farmer grows drought-tolerant crop varieties and 0 otherwise	0.55
Grow diversified crop	Dummy, 1 if the farmer engages in crop diversification and otherwise	0.34
Early maturing crop	Dummy, 1 if the farmer grows early maturing crop varieties and 0 otherwise	0.22
Income diversification	Dummy. 1 if the farmer engages in income diversification and 0 otherwise	0.18
Food-secure (outcome)	Dummy, 1 if the farmer is food secure and 0 otherwise	0.44
**Independent Variables**		
Gender	Dummy, 1 if the farmer is male and 0 otherwise	0.70
Age	Age of the farmer in years	53.24
Farm experience	Farming experience of the farmer in years	24.95
Education	Education level of the farmer in years	7.60
Living with spouse	Dummy, 1 if the farmer lives with a spouse and 0 otherwise	0.79
Household size	Number of family members in the farmer’s household	5.09
Agriculture	Dummy, 1 if the farmer’s income source is agriculture only and 0 otherwise	0.69
Land size	Total farm size owned by the farmer in hectares	2.23
Livestock	Number of livestock owned by the farmer’s household in Tropical Livestock Unit (TLU)	1.46
Member	Dummy, 1 if the farmer is a member of cooperative and 0 otherwise	0.33
Training	Dummy, 1 if the farmer participates in farmer training and 0 otherwise	0.21
Extension	Number of contacts with extension agents per month	0.27
Information	Dummy, 1 if the farmer regularly receives information on expected rainfall and temperature and 0 otherwise	0.70

The majority of the sampled farmers (70%) were male, with an average household size of 5.09. The average age of the farmers was 53.24 years old, with 7.60 years in schooling and 24.95 years in farming. On average, approximately 79% of the farmers lived with a spouse. Agriculture was the only income source for most farmers (69%) in the study area.

The average size of land held by the surveyed farmers was 2.23 ha. Many sampled farmers practiced integrated farming systems (a mix of crop cultivation and livestock rearing). The mean number of livestock owned by the sampled farmers’ households was 1.46 (as measured in the TLU). Approximately 33% of the sampled farmers reported that they were a member of agricultural input supply cooperatives. More than 20% of the sampled farmers had participated in training on farming. The number of extension agent contact an average farmer had per month was 0.27 times. The majority of the surveyed farmers (70%) reported that they had regularly received updated information on expected rainfall and temperature.

## 4 Empirical results and discussion

### 4.1 Adoption of climate change adaptation strategies (multivariate probit estimates)

Table 2 presents the results of the multivariate probit estimation of the determinants of the farmers’ climate change adaptation strategies, including the use of drought-tolerant crop varieties, growing diversified crops, use of early maturing crop varieties, and income diversification. These four dependent variables are assumed to be mutually inclusive, which means a farmer could use a combination of more than one climate-risk-copping strategy in the study area. A multivariate probit model is suitable to estimate these variables. A set of independent variables are included in the multivariate probit model based on microeconomic theory and a review of relevant literature.

**Table 2:** Determinants in the farmers’ adoption of climate change adaptation strategies (multivariate probit estimates).

**Variables**	**Use of drought tolerant varieties**	**Crop diversification**	**Early maturing crop varieties**	**Income diversification**
Gender	0.496 **(0.186)	0.124*(0.222)	−0.045(0.236)	0.226(0.271)
Age	−0.020**(0.007)	−0.013*(0.008)	−0.010**(0.009)	−0.003**(0.011)
Farm experience	0.024(0.006)	0.008(0.008)	0.009**(0.009)	(0.020**(0.011)
Education	0.009**(0.018)	0.054***(0.021)	0.020**(0.024)	0.027**(0.025)
Living with spouse	−0.262(0.203)	−0.025(0.247)	0.041(0.263)	0.095(0.314)
Household size	0.059**(0.028)	0.072**(0.030)	0.033(0.035)	0.001**(0.038)
Agriculture	0.190(0.313)	0.978**(0.481)	0.0278**(0.125)	−0.094(0.370)
Training	0.088***(0.329)	0.965**(0.490)	0.409**(0.124)	0.179**(0.385)
Livestock	0.048(0.042)	0.049(0.049)	0.055(0.051)	0.058**(0.059)
Member	0.260(0.132)	−0.060(0.164)	−0.160(0.179)	0.206***(0.187)
Land size	0.005(0.018)	0.013**(0.019)	0.072**(0.042)	0.022(0.027)
Extension	0.008(0.062)	0.137**(0.115)	0.229**(0.176)	0.047(0.084)
Information	0.197***(0.148)	0.385***(0.162)	0.144***(0.185)	0.095(0.195)
Constant	1.270(0.547)	3.300***(0.745)	0.932***(0.367)	0.785(0.720)
Cross equation Correlation	ρ_12_0.131*** (2.57)	ρ_13_0.140*** (2.14)	ρ_23_0.114*** (1.97)	ρ_33_0.127*** (2.78)
LR Chi-Square	127.21			
Prob > Chi-Square	0.000			
Number of observations	540			

Note: Standard errors are reported in parentheses. The results are significant at ***1%, **5% and *10% levels, respectively.

The results of the analysis show that the gender of the farmer is positively associated with the use of drought-tolerant crop varieties and crop diversification. The positive relationship between the average male-headed farmer and climate change adaptation strategies has been similarly found in other studies (Deressa et al., 2009, Aryal et al., 2020). Some studies found that male-headed farmers were less likely to adopt coping strategies against climate hazards than female-headed farmers (Nhemachena & Nhem, 2007), while other studies saw the gender of the farmers as insignificant (Ali & Erenstein, 2017). The age of the farmers is negatively associated with all four adaptation strategies, indicating that younger farmers are more likely to adopt these strategies to minimize the impact of climate change on food security. Younger farmers could be more receptive to recent agricultural innovations and keener to adopt new technologies and methods to improve their agricultural practices. The negative relationship between the farmers’ age and climate change adaptation strategies has been similarly found in other studies (Ali and Erenstein, 2017, Jamshidi et al., 2020). Farming experience positively influences the farmers’ decision to adopt early maturing crop varieties and engage in income diversification. The farmer’s education level is positively associated with all four climate change adaptation practices. Educated farmers are more likely to be aware of climate change and agricultural innovations and may be more interested in adopting new technologies and practices to adapt to climate challenges. Other studies have reported the positive relationship between education and adoption of climate risk management measures (Thomas et al., 2007, Hassan and Nhemachena, 2008, Deressa et al., 2009, Bryan et al., 2013, Abid et al., 2015, Ali and Erenstein, 2017, Jamshidi et al., 2020).

Household size is positively associated with the use of drought-tolerant crop varieties, crop diversification, and income diversification. Other studies have reported similar results in Kenya (Kabubo-Mariara & Mulwa, 2019). Doing farming as the only source of livelihood is positively associated with crop diversification and the use of early maturing crop varieties. Participation in farmer training is positively associated with all four types of adaptation strategies. Being a member of the input supply cooperative is positively associated with income diversification strategy. Being a member of cooperatives can help increase the farmers’ incomes through grain sales. The positive relationship between institutional membership and climate risk management strategies has been reported by Adesina et al., 2000, Ali and Erenstein, 2017, and Aryal et al. (2020). The mean land size of the surveyed farmers is positively associated with crop diversification and the use of early maturing crop varieties. The average landholding size among smallholder farmers in Kenya is as small as less than two hectares (FAO, 2015). Given their small landholding size, crop diversification and growing early maturing crops could be among the strategies for the farmers to reduce the adverse effects of climate change on their food production. This result is in line with the generally reported positive association between farm size and technology adoption (Tiwari et al., 2009, Bryan et al., 2013, Abid et al., 2015) as well as between farm size and climate change adaptation strategies (Ali and Erenstein, 2017, Kabubo-Mariara and Mulwa, 2019, Jamshidi et al., 2020). Farmers with large landholdings tend to have more financial capacity to try and invest in climate adaptation strategies. As land is a proxy for wealth, farmers with larger landholdings tend to adopt more climate adaptation strategies because of their financial ability to invest in new technologies and methods to adapt to climate change. The frequency of extension contact is positively associated with crop diversification and the use of early maturing crop varieties. This finding is in line with studies by Deressa et al., 2009, Ali and Erenstein, 2017. Regular access to information on expected rainfall and temperature are positively associated with the use of drought-tolerant crop varieties, crop diversification, and the use of early maturing crop varieties. Accessing information enables farmers to adopt such climate adaptation strategies (Abid et al., 2015, 2016). The cross-equation correlations are positive and significant at the 1% significance level, suggesting that these equations should be estimated jointly. The LR-Chi-square is also highly significant at the 1% significance level, indicating the robustness of the variables included in the model.

### 4.2 Number of climate change adaptation strategies adopted by the farmers (CLAD estimates)

Table 3 presents the determinants of the aggregate number of climate change adaptation strategies considered in this study using the Censored Least Absolute Deviation (CLAD) estimation. Results indicate that male farmers undertake more adaptation strategies compared to their female counterparts. Age is negatively associated with the number of climate change adaptation practices, indicating that younger farmers are more aware of climate change risks and related adaptation strategies than older farmers. This is possible because young farmers in Africa are generally aware of the risks of climate change and its adaptation and coping strategies (Ali and Erenstein, 2017, Jamshidi et al., 2020). The educational level of the farmers is positively associated with the number of climate adaptation strategies adopted, indicating that farmers with more years of education adopt more adaptation practices than those with fewer years of education. This is possible because the more educated farmers could be more aware of the risks of climate change and its adaptation strategies (Ali and Erenstein, 2017, Nor Diana et al., 2022). The positive and significant coefficient of farm household size indicates that the farmer living with a larger family size adopts more strategies to reduce climate change-related risks.

**Table 3:** Number of strategies adopted by the farmers (CLAD estimates).

**Variables**	**Coef.**	**t-values**
Gender of farmer (male = 1 & female = 0)	0.040**	0.15
Age of farmer	−0.014***	−0.44
Farming experience	0.009	0.94
Education of farmers (in years)	0.029***	1.11
Living with spouse	−0.050	−1.17
Household size	0.003***	0.03
Agriculture as the primary source of income	0.606***	1.33
If farmers participated in training	0.532***	1.20
Livestock (Tropical livestock units)	0.082	1.37
Member of cooperative	0.300	1.53
Land size in hectares	0.006**	0.20
Nos of contact per month with an extension	0.069***	0.71
If farmers receive regular information o weather	0.014***	0.07
Constant	0.009***	−0.22
Initial sample size	540	
Final sample size	403	
Value of *R^2^*	0.352	

The results are significant at ***1%, **5% and *10% levels, respectively.

The farmers whose occupation is solely farming adopt a greater number of adaptation practices compared to those who engage in both on-farm and non-farm activities. This result is possible because agriculture-dependent farmers could focus their investments on farming. Farmers with more hectares of land adopt a greater number of climate change adaptation strategies, possibly because they have a higher financial ability to invest in more climate change adaptation practices.

Participation in farmer training is positively associated with the number of climate change adaptation practices, suggesting that trained farmers acquire more knowledge and skills of different adaptation strategies related to climate change. The positive coefficient of extension contact indicates that the farmers who contact agricultural extension agents more frequently adopt a greater number of climate change adaptation practices. Farmers who receive information on expected rainfall and temperature on a regular basis adopt a greater number of strategies to reduce the risks of climate change extremes. These results suggest that more access to training, extension service, and information are critical in improving farmers’ knowledge and skills for adopting new technologies and practices related to climate change adaptation.

### 4.3 Impact of number of climate change adaptation strategies adopted on food security (PSM estimates)

Table 4 presents the effect of the number of adaptation strategies adopted by the farmers’ food security status based on the PSM analysis. Farmers who adopt one adaptation strategy have higher food security status (approximately 7–11%) compared to those who have not adopted, all the matching algorithms, including the nearest neighbor matching (NNM), radius (caliper) matching (RM), kernel-based matching (KBM), and stratification matching (SM).

**Table 4:** Effect of the number of climate change adaptation strategies on the farmers’ food security status (PSM estimates).

**Matching algorithm**	**Number of strategies**	**Outcome**	**ATT**	**t-values**	**Critical level of hidden bias**	**Number of treated**	**Number of controls**
NNM	1	Food- secure	0.07***	2.31	1.10 – 1.15	144	273
RMM			0.11***	1.58	1.20 – 1.25	152	266
KBM			0.10**	2.49	1.50 – 1.55	147	252
SM			0.08***	1.95	1.05–1.10	142	210
							
NNM	2	Food- secure	0.11*	1.53	1.25 – 1.30	127	245
RM			0.13**	1.41	1.45 – 1.50	141	248
KBM			0.14***	1.15	1.60 – 1.65	123	240
SM			0.10**	1.67	1.15 – 1.20	113	217
							
NNM	3	Food- secure	0.12**	1.53	1.35 – 1.40	112	243
RM			0.15***	2.08	1.20–1.25	127	226
KBM			0.14***	2.52	1.50 – 1.55	132	251
SM			0.13**	2.00	1.45 – 1.50	92	225
							
NNM	4	Food- secure	0.14***	1.98	1.35 – 1.40	120	197
RM			0.18***	3.14	1.15 – 1.20	91	155
KBM			0.17**	2.68	1.20–1.25	103	143
SM			0.15***	1.88	1.10–1.15	77	120

Note: ATT stands for the average treatment effect for the treated, NNM stands for the nearest neighbor matching, KBM stands for kernel-based matching, RM stands for radius (caliper) matching, and SM stands for stratified matching. The results are significant at ***1%, **5% and *10% levels, respectively.

Similarly, for all the PSMs, if the farmers adopt two adaptation strategies, their food security status is higher by 11–14% compared to those who have not adopted. If the farmers adopt three adaptation strategies, their food security status increases by approximately 12–15%. If the farmers adopt four adaptation practices, their food security status rises by approximately 14–18%. The positive relationship between the number of farmers’ climate change adaptation practices and their food security status was identified by Ali & Erenstein (2017) in Pakistan. Using NNM and KBM, Ali & Erenstein (2017) found that, if farmers adopted one adaptation practice, their food security status would increase by 7–8% compared to those who had not adopted any adaptation practice; if they adopted two or three adaptation practices, their food security status would increase by approximately 8–9% or 12–14%, respectively.

**Fig. 3:**
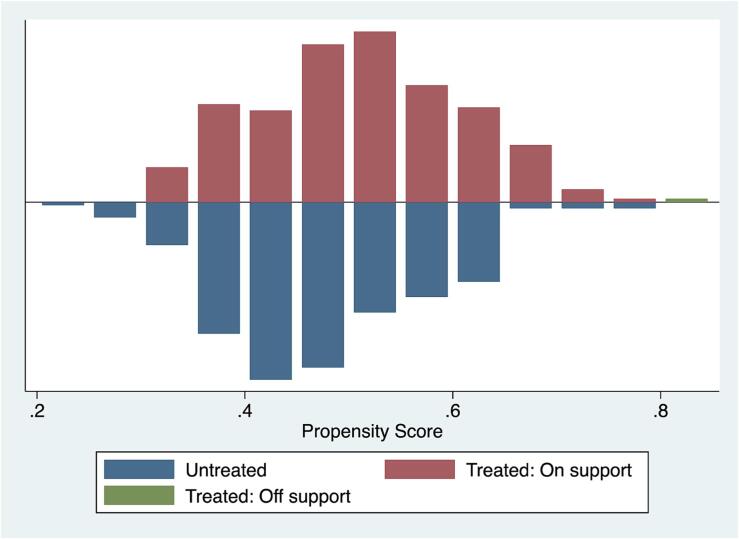
Propensity scores matching estimates.

Table 5 reports the critical level of hidden bias, indicating how much the farmers who adopted climate change adaptation strategies and those who did not differ from each other due to unobserved characteristics or odds of their adoption. Fig. 3 also graphically shows the indicators of covariates balancing, the imposition of the common support condition, and the balancing of covariates before and after matching, as presented in Table 5. As the main purpose of the PSM is to balance the covariates before and after matching, we employed a number of matching tests. Before matching, the median absolute bias was relatively high (10.02–28.76), but after matching, it was reduced to 3.43–9.87. The percentage of bias reduction is in the range of 65.67–76.51.

**Table 5:** Indicators of covariate balancing (before and after matching).

**Matching algorithm**	**Number of strategies**	**Outcome**	**Median absolute bias before matching**	**Median absolute bias after matching**	**% of bias reduction**	**Value of *R^2^* before matching**	**Value of *R^2^* after matching**	**Joint significance of covariates before matching**	**Joint significance of covariates before matching**
NNM	1	Food secure	18.50	4.43	76.51	0.461	0.002	0.002	0.435
RM			16.43	5.70	68.70	0.286	0.000	0.001	0.321
KBM			15.70	3.60	75.60	0.351	0.001	0.001	2.752
SM			10.02	3.43	71.04	0.297	0.000	0.001	1.921
									
NNM	2	Food secure	21.34	5.50	70.80	0.352	0.004	0.002	2.891
RM			19.32	4.00	72.50	0.220	0.001	0.000	3.725
KBM			17.22	6.32	65.67	0.274	0.003	0.002	5.210
SM			14.91	5.41	69.02	0.255	0.004	0.001	3.660
									
NNM	3	Food secure	24.50	8.89	70.00	0.297	0.005	0.000	4.431
RM			22.31	7.54	68.50	0.178	0.003	0.001	1.762
KBM			20.70	6.33	69.00	0.200	0.006	0.002	2.710
SM			19.20	7.20	65.00	0.173	0.002	0.000	1.671
									
NNM	4	Food secure	28.76	9.87	73.72	0.321	0.003	0.001	3.230
RM			19.10	4.56	70.45	0.196	0.001	0.000	2.190
KBM			23.85	8.30	67.88	0.212	0.002	0.001	1.520
SM			20.32	5.72	70.21	0.251	0.005	0.000	2.190

Note: NNM stands for the nearest neighbor matching, KBM stands for the kernel-based matching, RM stands for radius (caliper) matching, and SM stands for stratified matching.

Similarly, the value of *R^2^* is quite high before matching (0.173–0.46), but it is quite low after matching (0.000–0.004), signifying that, after matching, both groups are very similar to each other. The p-value of joint significance of covariates is significantly lower before matching, but higher after matching. This indicates that, before matching, both categories of the farmers (adopters and non-adopters) are significantly different, but after matching, they are quite similar to each other.

## 5 Conclusion and policy implications

We examined the factors affecting the surveyed farmers’ choice of climate change adaptation strategies and associated effects on their food security in Kenya. Farmers in Kenya are using different adaptation strategies to counter the negative impacts of climate change. Our survey with 540 farmers from across six counties of Kenya in 2018 found that approximately 55% of the farmers adopted the planting of drought-tolerant crop varieties, about 34% a crop diversification strategy, roughly 22% the planting of early maturing crop varieties, and about 18% an income diversification strategy.

The results of the multivariate probit model suggest that male-headed farmers are more likely to use drought-tolerant crop varieties and crop diversification to cope with climate risk. Younger farmers and those with higher levels of education are more likely to use these climate change adaptation strategies. The farmers with larger land sizes and solely reliant on agriculture as their livelihood source have significantly positive associations with crop diversification and the use of early maturing crop varieties. Participation in farmer training, membership in input supply cooperatives, contact with extension agents, and access to information on expected rainfall and temperature are positively associated with different adaptation practices adopted by farmers in the survey area. Access to training, extension services, and information would be critical in improving farmers’ knowledge and skills for adopting new agricultural technologies and practices related to climate change adaptation in the study area.

Results of the CLAD analysis suggested that the male-headed farmers, education level, family size, land size, farm income, extension contact, access to training, and access to information are positively associated with the number of adaptation strategies adopted by the farmers in the survey area. The results of the multivariate probit and CLAD estimation revealed some interesting patterns, which are unique in the context of Kenya, with significant policy implications. Firstly, the results highlight the importance of farmers’ knowledge and awareness about the local context, climate change adaptation strategies, and their benefits. Secondly, the results point to the importance of wealth on the ability of the farmers to invest in climate adaptation strategies. Hence, policy should focus on two aspects: (i) increasing farmers’ awareness of climate change and potential benefits from adopting climate change adaptation strategies; and (ii) increasing farmers’ capacity for climate change adaptation by augmenting their assets (e.g., land, education, extension advice and training, farm income) while controlling the cost of adaptation. The policy on increasing farmers’ awareness should focus on increasing their access to education and agricultural extension services. The policy on enhancing farmers’ accessibility to climate change adaptation strategies should focus on increasing their endowments, for instance, by improving the government provision of extension services, farmers’ participation in farmer training, their access to information on expected rainfall and temperature, and creating opportunities for their alternative livelihoods, such as non-farm activities. Such support should be provided especially to economically less privileged farmers and women.

The results of the PSM analysis show an increasingly positive relationship between the number of climate change adaptation strategies adopted by the surveyed farmers and their food security status. This finding has important policy implications. Government of Kenya and other relevant organizations should encourage farmers to adopt drought-tolerant varieties, crop diversification, early maturing crop varieties, and income diversification as part of their extension strategy to have them adapt to climate change and improve their food security status. The extension to farmers should emphasize that adopting more climate adaptation practices will generally help improve their food security.

A major limitation of this study is related to its focus on the use of cross-sectional data to gauge the effect of farmers’ climate change adaptation on food security. We suggest that future studies on the effect of climate change adaptation on food security should be based on data collected over multiple periods, given that climate change effects vary over time. Second, where the database allows, there is a need to conduct a comparative analysis across regions in Kenya, as climate change has different effects across regions.

## Declaration of Competing Interest

The authors declare that they have no known competing financial interests or personal relationships that could have appeared to influence the work reported in this paper.

## Data Availability

The data that has been used is confidential.

## Footnotes

1 *An expected treatment effect on the treated is the difference between the outcome of the treated observations and that of the hypothetical observations had they not been treated.*
